# δ^15^N Value Does Not Reflect Fasting in Mysticetes

**DOI:** 10.1371/journal.pone.0092288

**Published:** 2014-03-20

**Authors:** Alex Aguilar, Joan Giménez, Encarna Gómez–Campos, Luís Cardona, Asunción Borrell

**Affiliations:** Department of Animal Biology and Biodiversity Research Institute (IrBio), Faculty of Biology, University of Barcelona, Barcelona, Spain; Texas A&M University-Corpus Christi, United States of America

## Abstract

The finding that tissue δ^15^N values increase with protein catabolism has led researchers to apply this value to gauge nutritive condition in vertebrates. However, its application to marine mammals has in most occasions failed. We investigated the relationship between δ^15^N values and the fattening/fasting cycle in a model species, the fin whale, a migratory capital breeder that experiences severe seasonal variation in body condition. We analyzed two tissues providing complementary insights: one with isotopic turnover (muscle) and one that keeps a permanent record of variations in isotopic values (baleen plates). In both tissues δ^15^N values increased with intensive feeding but decreased with fasting, thus contradicting the pattern previously anticipated. The apparent inconsistency during fasting is explained by the fact that a) individuals migrate between different isotopic isoscapes, b) starvation may not trigger significant negative nitrogen balance, and c) excretion drops and elimination of ^15^N-depleted urine is minimized. Conversely, when intensive feeding is resumed in the northern grounds, protein anabolism and excretion start again, triggering ^15^N enrichment. It can be concluded that in whales and other mammals that accrue massive depots of lipids as energetic reserves and which have limited access to drinking water, the δ^15^N value is not affected by fasting and therefore cannot be used as an indicatior of nutritive condition.

## Introduction

The use of stable isotopes to study animal ecology and physiology has experienced an explosive growth in the last twenty years until becoming a technique of choice to investigate habitat use, migration, diet, physiology and nutritive condition (e.g. [1]–[3]). Among the many useful elements, nitrogen has received extensive attention because the proportion between its two stable isotopes varies in a consistent and predictable manner following well-defined processes such as trophic transfer and protein catabolism. Because each isotope has different atomic mass, the nitrogen physiological pathways affect them in a different manner: The lighter isotope (^14^N) is more readily excreted through urine than the heavier isotope (^15^N). Such dissimilar excretion rate leads to a long-term increase from prey to predator of the ratio ^15^N/^14^N, which is commonly expressed as the δ^15^N value (see methods).

In addition, it has been hypothesized that during the periods of extreme nutritional stress in which the organism resorts to protein catabolism to obtain energy, such as during prolonged fasting leading to starvation, the negative nitrogen balance and the differential excretion of the two isotopes triggers the depletion of the lighter ^14^N in the free amino acid body pool [4]. Since the only source of nitrogen to build-up new protein or restore protein-rich tissues is precisely that transported by serum, the loss of lighter nitrogen as a result of excretion causes the δ^15^N value of residual amino acids to increase. When rebuilding of protein is made in the absence of exogenous nitrogen sources, the tissue will be necessarily constructed with these ‘heavy’ amino acids, therefore eliciting an increase in its δ^15^N values [5], [6].

Taking this mechanism as a basis, the δ^15^N value has been proposed as an index of nutritional condition that summarizes the energetic balance of an organism [4]. After the merit of this fasting-increasing hypothesis was confirmed in a number of species that ranged from insects to mammals, including humans [2]–[4], [7]–[13], it became generally accepted that it might occur in any potential species and scenario. Today, standard interpretation of isotopic results take for granted that catabolism of protein from tissues leading to severe negative introgen balance often results in an increase in the δ^15^N value of the body nitrogen pool (e.g. [3]). However, the phase in the starvation process in which the body of homeotherms resorts to significant catabolism of lean body tissue only occurs when fat reserves are nearly exhausted and the individual is shortly approaching death [14]–[15]. As a consequence, it is not surprising that during the last decade some studies have come into apparent conflict with the above hypothesis [16], particularly when the species involved was a capital breeder that was fasting not only for food but also for water. For example, marine mammals, which mostly rely on food as the main water source [17], have produced recurrent examples of dissension [18]–[21].

To elucidate the actual long-term changes experienced by the δ^15^N value in animals subject to periodical cycles of intensive feeding and fasting in absence of substantial water intake, we investigated nitrogen isotopic dynamics in the fin whale *Balaenoptera physalus*. Adequate material for this study was available because this species had been exploited off Spain until 1985 and tissue samples of individuals of known age, sex and reproductive state had been collected as part of the biological monitoring of the population and preserved in a tissue bank. As in most mysticetes, this species alternates summer high-latitude destinations, where individuals feed intensively, with winter low-latitude destinations, where individuals find the warm environment necessary to reproduce but are forced to fast because food is scarce or non-existent [22]. We analysed two tissues providing complementary insights: i) the muscle of individuals sampled throughout the summer feeding season, which is expected to integrate diet over some months prior to collection [23], and ii) the baleen plates, structures that grow continuously and, being composed of metabolically inert tissue, do not experience isotopic turnover. Because of this, baleens keep a permanent record along their length of the isotopic signal in the nitrogen body pool at the time of tissue formation. Accordingly, baleen plates have been frequently used to investigate movement patterns or temporal shifts in diet [21], [24]–[30].

As opposed to what would be expected according to the fasting-^15^N enrichment hypothesis, δ^15^N values markedly decreased during the fasting season, coincidentally with the mobilization of body reserves. Conversely, when food intake was re-established, δ^15^N values increased until reaching previous values, again as opposed to currently accepted understanding.

## Methods

### Ethical statement

The samples were obtained from whales subject to legal commercial exploitation in the 1980s and preserved thereafter at the BMA Tissue Bank of the University of Barcelona. In Spain, no specific permits are required to carry out research on samples stored at public institutions.

### Sample collection and preparation

The samples were collected in the 1983–1985 summer seasons at the Caneliñas whaling station (NW Spain). The muscle tissue was collected from the trunk region, posterior to the dorsal fin, and preserved at –20°C. Date of capture and biological data were available from each individual. To avoid potential interference of reproduction on stable isotopic values [3], only males (N = 55; 45 sexually mature, 10 sexually immature) were selected for the study. From each sample, a subsample of 1 g of muscle was dried for 48 h at 60°C and powdered with mortar and pestle.

Baleen plates were collected from five individuals identified as A, B, C, D and E. Surface oils and adhered material were removed using a steel palette knife, steel wool and a chloroform:methanol solution (2:1), and stored dry until analysis. No information on date of capture or biological characteristics was available. To allow comparison with previous studies on fin whales [29]–[30], subsamples were extracted with a grinder every 1 cm along the length of each plate until totaling 40 subsampling points.

### Stable isotope analysis

For both tissues, approximately 0.3 mg of powdered sample were weighed into tin capsules, automatically loaded and combusted at 1000°C. in a continuous flow isotope ratio mass spectrometer (Flash 1112 IRMS Delta C Series EA; ThermoFinnigan, Bremen,Germany). Analyses were performed at the Centres Científics I Tecnològics (CCiT-UB) of the University of Barcelona (Barcelona, Spain). The stable isotope N ratio was expressed in delta (δ) notation, while the relative variation of stable isotope ratio was expressed as permil (‰) deviations from the predefined international standards as:
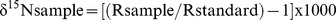



where Rsample and Rstandard are the ^15^N/^14^N ratios in the sample and standard, respectively. The standard used was nitrogen (air) for ^15^N. International secondary standards provided by the International Atomic Energy Agency (IAEA, Vienna, Austria) were inserted every 12 samples to calibrate the system and compensate for any drift over time. The precision and accuracy for δ^15^N measurements was 0.3‰ [31].

### Estimation of the growth rate of baleen plates

Because fluctuations of δ^15^N values in baleen plates are assumed to be caused by the migratory regime from wintering to summering grounds the length of plate occurring between two following minimum δ^15^N points was interpreted as the growth that had taken place during a complete cycle, *i.e.* one year.

### Statistical analysis

Data were tested for normality with the Shapiro-Wilk test and for heteroscedasticity with the Levene’s test. Differences between groups were investigated either with the U-Mann Whitney or with ANOVA, according to data normality. Relationships were analyzed through standard regression. All above tests were made with package SPSS 15.0. Fluctuations in baleen plates were gauged through a generalized additive model (GAM) fitting a semi-parametric regression with smoothing by cross-validation using R (R Development Core Team 2010).

## Results

### δ^15^N values in muscle

**Figure 1:**
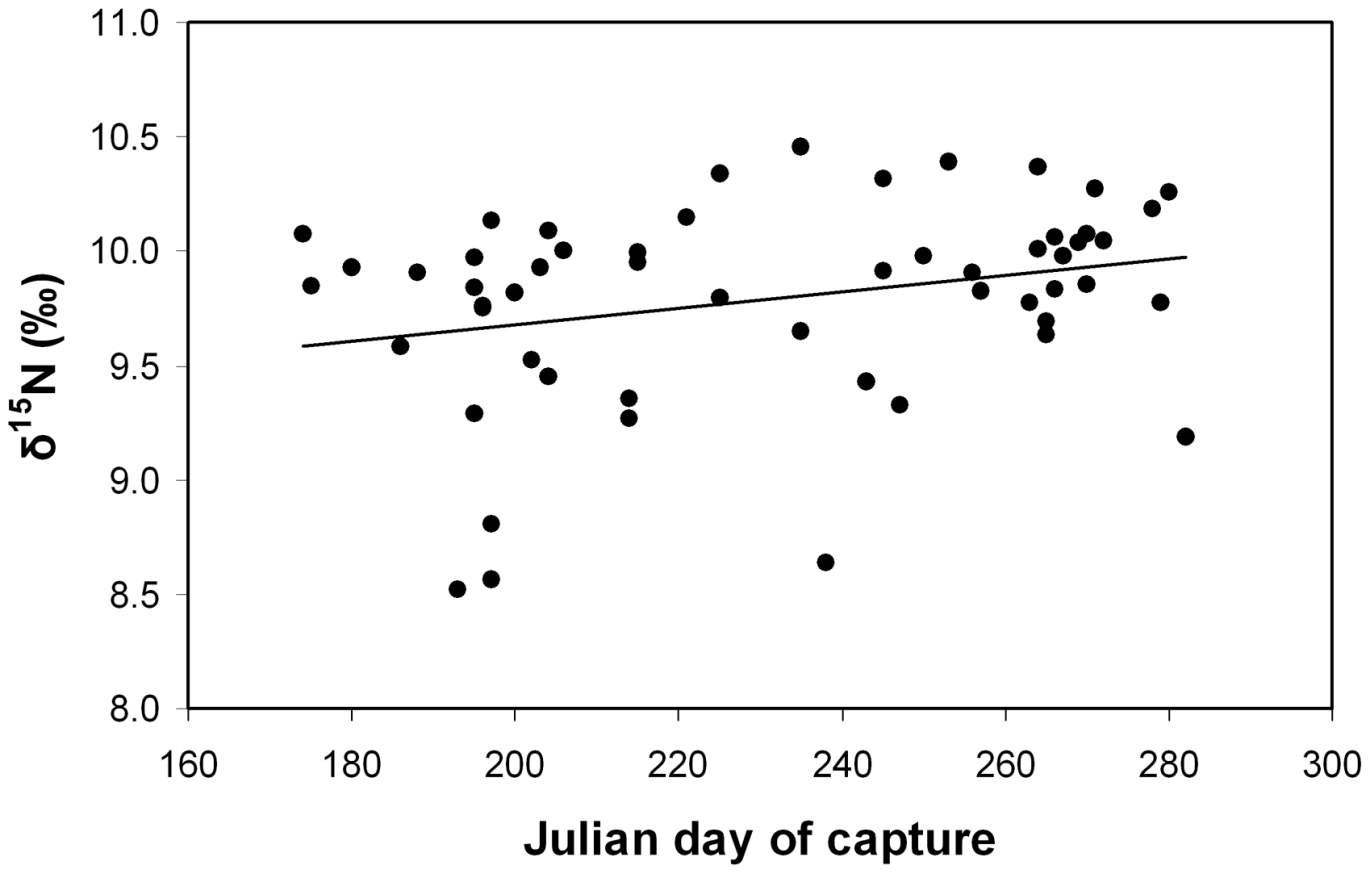
Relationship between the δ^15^N value in muscle and the Julian day in which the individual was sampled (r^2^ = 0.0729, p<0.05).

δ^15^N values in the individuals sampled are detailed in Table S1. The mean of δ^15^N values was 9.8‰ (Table 1). Significant differences were not detected between immature and mature males (U = 191, p = 0.458), so both groups were combined in the investigation of patterns of variation. δ^15^N values were regressed against the day of capture of each individual, which reflects the time spent at the feeding grounds, and a weak but positive statistically significant relationship was found (r^2^ = 0.0729, p<0.05; Figure 1).

**Table 1:** Mean ± standard deviation and range of the nitrogen stable isotope results in muscle and baleen plates (mean of all subsamples).

Tissue	Stable isotope	Average (‰) ± SD	Range (‰)	
Muscle	δ^15^N	9.8±0.44	8.5	10.4
Baleen plates	δ^15^N	9.9±0.50	8.5	10.7

### δ^15^N values in baleen plates

**Figure 2:**
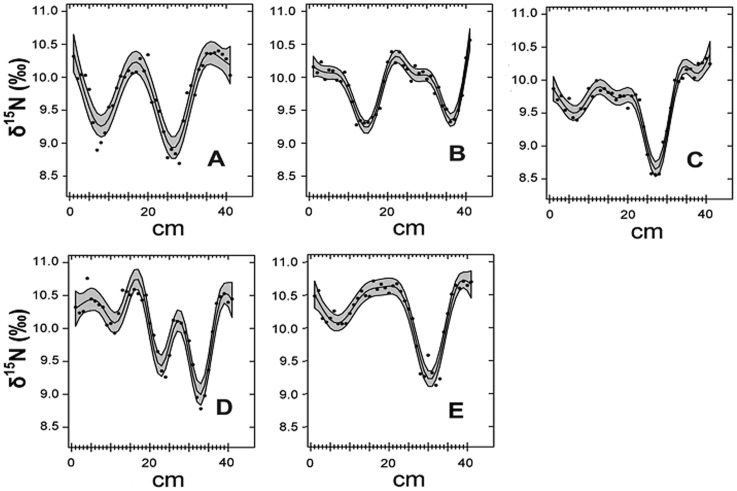
Variation of δ^ 15^N values along the growth axis of the baleen plates (measured in cm from the gum) from the five individuals analysed, identified as A-E. Points depict actual values; the continuous line depicts the fit of the GAM modelling; the blue fringe shows the 95% confidence intervals.

δ^15^N values measured in the various sampling points of each baleen plate are detailed in Table S2. The mean δ^15^N value for the five individuals combined was 9.9‰ (Table 1). Although an ANOVA showed statistically significant differences between individuals (F_4,200_ = 9.919, p<0.001), all baleen plates showed periodic fluctuations in δ^15^N values along their growing axis (Figure 2) with the unerupted section of the gum showing the highest δ^15^N value of the entire baleen plate. The mean amplitude of such fluctuations (δ^15^N max. – δ^15^N min.) was 1.1±0.47‰, with a maximum of 1.8‰. The distance between two following minimum δ^15^N value points was assumed to correspond to one year and therefore used to estimate the mean annual growth rate of the plate. The generalized additive model (GAM) fits showed that the amplitude was not homogeneous: despite small irregularities likely caused by individual variation in migratory or foraging behaviour, in four individuals (A, B, C and E) amplitude was very similar and cycles were completed every 18–23 cm (mean = 20 cm). Conversely, the fifth individual (D) showed a less defined pattern, with shorter spacing between minimum δ^15^N value points and cycles being completed every 11–12 cm. The reason for the dissimilar pattern of baleen plate D is unknown, but it is again likely to be caused by differences in the behaviour of this individual. As a consequence, results from baleen plate D were not included in the calculations of the mean fluctuations. Whatever the case, the mean amplitude of fluctuations did not show any significant relation with either the color nor the size of the baleen plate (Table 2, Figure 2).

**Table 2:** Characteristics and δ^15^N values of the baleen plates used in the analysis.

Individual	Length (cm)	Colour of baleen plate	δ^15^N(‰) ± SD	Interval between lower δ^15^N values (cm)
A	51	Dark grey	9.8±0.5	18
B	60	Grey	9.9±0.4	19
C	46	Yellow	9.7±0.4	20
D	58	Grey	10.1±0.5	11–12
E	48	Grey with yellow	10.2±0.4	23

## Discussion

The δ^15^N values of baleen plates showed a regular fluctuation pattern along their growth axis (Figure 2) comparable to that previously found in fin whales and other mysticetes and which are generally attributed to the migratory regime of the species and the associated alternation of intensive feeding and fasting [21], [24]–[30]. Like most other large mysticetes around the world, fin whales undergo annual latitudinal migrations which involve feeding seasonality. During the summer, they stay at high latitudes, where feeding grounds are located, while during the winter they migrate to lower latitudes to breed [22]. The northwestern coast of Spain, where the samples for this study were collected, is a feeding area for one of the fin whale stocks of the eastern Atlantic population. Individuals reach this area in spring after migrating from breeding grounds situated at a southern undetermined location and where they have fasted for a several-month period.

Life-cycle of fin whales involves profound changes in body composition. During the season of intensive feeding individuals consume massive amounts of food that contain equally massive amounts of surplus energy which are stored in the form of fat in the hypodermic cover, commonly called blubber [26], [27]. Food also provides preformed water that allows the individuals to avoid having to drink seawater, which contains salts that are energetically costly to excrete [17]. During fasting, energy requirements for vital functions, including reproduction and migration, must be supplied by these blubber reserves, although the lean tissue may also be mobilized to a smaller extent to produce glucose from amino-acids through gluconeogenesis or to rebuild essential proteins. Mobilization of lipids also serves to liberate endogenously-produced metabolic water, thus reducing ingestion of salty seawater to a minimal quantity, which in a number of whale species has been estimated at only 4.5–13 ml kg^-1^ day^-1^ [17], [34].

Based on the assumption that δ^15^N value fluctuations in the baleen plate are caused by the migratory regime, and omitting individual D, which showed a different fluctuation pattern than the other individuals, the mean annual baleen plate growth was estimated at 20.0 cm (SD: ± 2.16 cm), a value in accordance to previous estimations for the Mediterranean fin whale [29], [30] and consistent with that observed in other mysticetes such as the minke whale, *Balaenoptera acutorostrata* (12.9 cm/year) [27], the bowhead whale, *Balaena mysticetus* (20 cm/year or less) [35], [36] and the southern right whale, *Eubalaena australis* (27 cm/year) [21].

Considering this growth rate as well as the fact that the whales had been sampled in the summer while they were at the feeding ground, the un-erupted section of the baleen plate (the basal region) was inferred to have been formed when the individual had already arrived to the feeding area. According to this, its isotopic signature should correspond to that of the tissue built at the beginning of the period of intensive feeding. According to the same reasoning, the immediately following distal segments of the plate were taken as representative of tissue formed in the previous months, when feeding was just starting or, if going further distally along the plate, when the whale was fasting or when its food intake was low. The pattern of variation (Figure 2) shows that the δ^15^N values tended to increase as the feeding season advanced and to decrease when whales were fasting. The results obtained from the muscle pointed in the same direction: the δ^15^N values significantly increased with day of capture throughout the season of intensive feeding indicating that enrichment was occurring concomitant with the improvement of body condition.

This pattern is novel because previous findings in a variety of avian and mammalian species indicated exactly the opposite pattern, this is, that during the fasting period δ^15^N values increased or, at least, showed no definite variation. The enrichment was attributed to catabolism of protein reserves containing nitrogen that had already been enriched in ^15^N relative to diet and that, when subsequently used to rebuild new tissue, experienced a second, additional enrichment [4], [9], [37]–[39]. Conversely, the absence of definite variation in δ^15^N values despite the occurrence of fasting was explained by the existence in some species of biochemical and physiological pathways that allow energy production without protein mobilization and, thus, without additional fractionation of nitrogen [16], [19]. The latter is consistent with the fact that in fasting animals not all tissues lose nitrogen in the same degree and in the same way [40]. Thus, Martínez del Rio *et al.* [41] suggested that tissues that have significant synthesis while fasting may be more prone to increase their δ^15^N values, while tissues with no synthesis would keep their original isotopic value.

The results of the present study on fin whales point to a different, if not opposite, scenario. Indeed, this is not an exceptional finding in this group of mammals and previous studies on other mysticetes had already produced results that contradicted the enrichment-by-fasting hypothesis. Thus, peaks or enrichment that corresponded to the periods of intensive feeding had been previously observed in southern right whales [21], bowhead whales [24], [42], and North Atlantic right whales *Eubalaena glacialis* [43], but authors attributed the apparent anomaly to insufficient sample size, effect of migration or changes in diet. Thus, the enrichment of δ^15^N values associated to intensive feeding seems to be a norm in this particular group of animals, rather than an exception.

The apparent contradiction between the dynamics of δ^15^N values in baleen whales and those in other species may be explained by three facts which are not mutually exclusive and the effects of which may operate in the same direction and additively: a) that during migration whales move through different δ^15^N isoscapes, b) that starvation does not trigger in whales a negative nitrogen balance because the limited winter feeding might allow them to maintain that balance while failing to meet caloric requirements, and c) that excretion of nitrogen wastes drops during fasting, thus minimizing elimination of ^15^N-depleted urine.

The first explanation seems supported by the pattern of geographical variation in δ^15^N baselines. Although the precise location of the wintering grounds of the eastern North Atlantic fin whale population is unclear, it is undoubtedly situated south of the Iberian Peninsula, probably off the Gibraltar Straits, off the northwestern coast of Africa or in the southwestern Mediterranean Sea [44]–[45]. In the temperate eastern North Atlantic, fin whales mostly feed on the northern krill *Meganyctiphanes norvegica*, the diet of which is mainly based on copepods [46]–[47]. The δ^15^N values of zooplankton from the upper fringe of the ocean (0–500 m), primarily composed of copepods, range between approximately 4.5‰ in the putative winter grounds of the fin whale population to approximately 6‰ in its summer feeding grounds [48]. The fluctuations in δ^15^N values along baleen plates ranged on average 1.1‰, so they fit well that variation (1.5‰).

However, this does not appear to be the only factor behind the observed pattern of variation of δ^15^N values. Thus, the severe decline in energy body reserves occurring in the fin whale during the fasting season [33] indicates that, similarly to other mysticetes, the quantity of food ingested per day during that period would only likely amount about 10% or less of that consumed during the summer and would hence be under maintenance levels [32]. Tissue δ^15^N values are known to increase not only in fasting vertebrates, but also in those feeding below maintenance levels because the isotopic value of a small daily ration cannot compensate for the highly ^15^N-enriched serum resulting from the mobilization of muscle protein [11]. In spite of this, δ^15^N values in baleen plates did not increase during fasting as expected but, rather the contrary, they decreased.

Here comes into play the second explanation mentioned above, this is, that in baleen whales starvation may not trigger significant mobilization of lean body mass and, therefore, a negative nitrogen balance. This pattern has been observed in other marine mammals and it may therefore be equally expected in baleen whales [19], [49]–[51]. Indeed, the use of protein as a source of energy –a main reason for the observed δ^15^N increment in terrestrial species– is probably very limited in a fasting whale, if at all existing. As all marine mammals, mysticete whales have massive lipid reserves deposited in the blubber, a tissue of multifunctional nature that constitutes the external cover of the body and that represents up to 15–45% of the individual body mass [17], [52]. Thus, baleen whales and other marine mammals are able to mobilize massive amounts of lipids to sustain catabolism without compromising overall functions [33], [52], [53] and the wealth of energy that such mobilization produces likely allows protein conservation even during prolonged fasting. In fasting elephant seal pups, for example, protein catabolism contributes less than 4% to the metabolic rate [50], [54]. To this it should be added that the physiological phase of starvation during which only fat is mobilized (the so-called phase two in the starvation cycle [55]) is especially protracted in animals that recycle urea, such as deer and at least some marine mammals [15], , thus facilitating protein sparing [58]. Proteins become thus a fuel of last resort and the switch from lipid-dominated catabolism to protein-dominated catabolism would be delayed to an extremely advanced stage of starvation [58]. As a consequence of all this, and unless the individual confronts extreme starvation, the amount of lean body tissue mobilized along a standard migratory cycle would be meager and unlikely to elicit the δ^15^N increase that is typically associated with fasting in terrestrial vertebrates [4], [9], [38]. In this situation, a little consumption of protein-rich zooplankton during the fasting period would provide the basic requirements for protein recycling and would therefore preserve body nitrogen balance. In other words, whales would be energy-starved, but not protein-starved.

The third potential explanation to explain the observed variation in δ^15^N values is that, differently to most terrestrial mammals, marine mammals have severely limited access to non-salty water during fasting because the main source of preformed water in these animals is food [17], [34]. In this particular physiological scenario, the compromise for water would appear parallel or even earlier than the compromise for energy. When this happens, blubber would become the main source of both calories and metabolic water [17]. Parallel to this, the activity of the kidney would be reduced and the resorption of endogenous electrolytes and body water would increase to help maintaining the homeostasis of fluids and electrolytes [34]. This would result in a minimization of water loss through defecation or urination to the smaller indispensable, probably to only 10–15% of normal values similarly to what has been observed in other mammals deprived of water during fasting [50], [59], [60]. If this is so, mysticete whales should have mechanisms, like hibernating bears or fasting postweaned elephant seals have, to avoid toxic concentrations of urea resulting from catabolism and they may have developed the ability to recycle urea by channelling their nitrogen to amino acid formation, in this way contributing to preserve lean body mass [57], [61].

It should be noted that these are completely different conditions from those faced by terrestrial herbivores that live in dry environments, which tend to show higher δ^15^N values than comparable species inhabiting wetter environments [62]–[65]. In these animals, the restriction in water intake is not associated with a parallel restriction in food intake and, therefore, recycling nitrogen is not a main requirement for them but, rather the contrary, their prime necessity is to get rid of nitrogen wastes with minimal water loss, towards which end they excrete highly concentrated, ^15^N-depleted urine. Also, nitrogen discrimination tends to be higher in herbivores than in carnivores, thus eliciting ^15^N enrichment [66]. Therefore, parallelism cannot be claimed to interpret variation in δ^15^N values in whales. Moreover, the effect of water-stress conditions on δ^15^N value appears to be currently disputed and indeed attributed to denitrification processes in the soil of dry environments that directly affect the isotopic values of the plants which compose the diet of their consumers [67]. Also, many of the species inhabiting such environments are ruminants [63], and a further increase in δ^15^N values is likely to be expected in them because they recycle urea into the rumen to support microbe growth. When this happens along successive cycles, the assimilation of nitrogen by microbes and the subsequent digestion of these microbes are likely to bring an increase in δ^15^N values through a process comparable to that occurring with trophic levels [68]. Finally, a further confirmation that fasting whales do not adjust to this physiological scheme, and that excretion of nitrogen is not a pressing need for them, appears to be confirmed by the fact that, contrary to the water-conservation mechanism of concentrating urine observed in many other groups of mammals, fasting cetaceans produce a diluted urine [34], [69]–[71].

Accordingly, newly formed tissue in the baleen plate of a fasting fin whale would reflect the δ^15^N signal from the scant food eaten in the wintering areas and also the shift to an energetic system based almost only on the consumption of body lipids to fulfill two physiological objectives: sparing muscle, which is necessary for migration, and obtaining metabolic water, a critical necessity in a fasting marine mammal because drinking a lot of sea water would imply the elevated energetic costs involved in salt excretion [17]. ^15^N-enrichment would not occur simply because severe negative nitrogen balance, the main driver of the process, is avoided even during fasting.

When whales reach the summer grounds and start intensive feeding, fat catabolism would end and protein anabolism would resume. Also, because preformed water would be more available through feeding, the activity of the kidney and the urine flow rate would raise, thus allowing for increased excretion of nitrogen and electrolytes [34]. The ^15^N enrichment observed to occur both in muscle and in baleen plates during the season of intensive feeding would therefore be again the result of at least three processes: the ingestion of food in a isoscape characterized by a δ^15^N value higher than that consumed during the winter period of low food intake, the resumption of high rates of N excretion -with the concomitant discrimination and enhanced elimination of ^14^N from the body pool-, and the increased intensity in feeding on a protein-rich diet, a fact that is commonly associated with a rise in the δ^15^N value of the nitrogen body pool [3], [72].

It can be concluded that in mysticetes, and probably also in other mammals that accrue massive depots of lipids as energetic reserves and which have limited access to drinking water, fasting does not induce negative nitrogen balance. As a consequence, ^15^N enrichment does not take place and the δ^15^N value cannot be used as indication of nutritive condition. Irrespective of the physiological implications of the δ^15^N variations observed, the amplitude of the fluctuations observed in the baleen plate bear also practical consequences on the use of this tissue to infer diet composition. The range of variation (about 1.1±0.5‰ in average and reaching a maximum of 1.8‰) correspond to a variation of about 0.46 trophic levels, a much higher value than the effect of fasting on seabirds [73]. As a consequence, although stable nitrogen isotopes still provide a useful measurement of trophic level in baleen whales, the disruptive effect of feeding and fasting, and the potential variation in isotopic baselines at the various locations visited during migration, should be incorporated into any diet-related analysis.

## Supporting Information

Table S1 Biological information of the individuals sampled and δ^15^N values measured in their muscle**.**
(XLSX)

Table S2 δ^15^N values measured in the various sampling points of the baleen plates.
(XLSX)
